# Histopathology of murine toxoplasmosis under treatment with dialyzable leukocyte extract

**DOI:** 10.1590/0074-02760170045

**Published:** 2017-11

**Authors:** Beatriz Eugenia Fuentes-Castro, Juan Gerardo Reyes-García, María Teresa Valenzuela-Vargas, Federico Martínez-Gómez

**Affiliations:** 1 Instituto Politécnico Nacional, Escuela Nacional de Ciencias Biológicas, Departamento de Parasitología, Ciudad de México, México; 2 Instituto Politécnico Nacional, Escuela Superior de Medicina, Sección de Estudios de Posgrado e Investigación, Ciudad de México, México; 3 Instituto Politécnico Nacional, Escuela Nacional de Ciencias Biológicas, Departamento de Morfología, Ciudad de México, México

**Keywords:** transfer factor, dialyzable leukocyte extract, toxoplasmosis, Toxoplasma gondii, histopathology

## Abstract

**BACKGROUND:**

Dialyzable leukocyte extracts (DLEs) contain molecules smaller than 10 kDa with biological activity in receptor organisms. Primarily, they participate in the regulation of the Th1 immune response, which is essential for the control of several intracellular infections, such as toxoplasmosis. This disease is associated with congenital infection, encephalitis or systemic infections in immunocompromised individuals. The clinical course of this infection fundamentally depends on a well-regulated immune response and timely treatment with the appropriate drugs.

**OBJECTIVE:**

The aim of this study was to evaluate the effect of treatment with a leukocyte extract, derived from crocodile lymphoid tissue, on the histopathology and brain parasite load in NIH mice that had been infected with cysts of *Toxoplasma gondii* (ME-49 strain).

**METHODS:**

The treatment was applied during the acute and chronic stages of the infection. Histopathological changes were evaluated in the ileum, liver and spleen at one, four and eight weeks after infection and in the brain at week 8. The parasite load was evaluated by counting the cysts of *T. gondii* found in the brain.

**FINDINGS:**

Compared to the control mouse group, the mice infected with *T. gondii* and under treatment with DLE showed less tissue damage, mainly at the intestinal, splenic and hepatic levels. In addition, a greater percentage of survival was observed, and there was a considerable reduction in the parasite load in the brain.

**CONCLUSIONS:**

The results suggest that DLE derived from crocodile is a potential adjunctive therapy in the conventional treatment of toxoplasmosis.

Dialyzable leukocyte extracts (DLE), or transfer factors, are defined as immunomodulatory molecules with molecular weights of approximately 10 kDa; they are derived from individuals sensitised to a particular antigen and have the property of transferring specific cell-mediated immunity to individual recipients (Lawrence 1955, Kirkpatrick 2000). Leukocyte extracts are commercially sold and can be obtained from human blood (Espinosa-Padilla et al. 2009), bovine dialyzable leukocyte extract and bovine colostrum (Sánchez-González et al. 2011), egg yolk (Xu et al. 2006), and crocodile spleen (Merchant et al. 2006, Theansungnoen et al. 2014). In research laboratories, these extracts are obtained from mice exposed to specific antigens (Fabre et al. 2004).

The effects of these extracts may involve activation of signalling pathways, cellular factors and transcription factors, as well as regulation of the Th1 response. Successful clinical applications have included those in patients with tuberculosis, Herpes simplex, asthma and some cancers (Fabre et al. 2004, Franco-Molina et al. 2008, Espinosa-Padilla et al. 2009, Rodríguez-Flores et al. 2015). In Parasitology, the use of DLEs has proven an effective treatment for Leishmaniosis and Echinococcosis, improving the clinical profile and immune response of most recipient patients (Delgado et al. 1981, Dvoroznáková et al. 2009).

The immune system of crocodilians has not been well characterised, but there are several reports that describe moderate antimicrobial activity. The protective effect of crocodile-derived serum, plasma and crude leukocyte extracts on bacterial, fungal and viral infections has been evaluated, and each of these provided different degrees of protection. These protective activities were attributed to the serum complement protein system and the presence of antimicrobial peptides of leukocyte extracts (Merchant 2006). The serum and leukocyte extracts have shown the greatest benefits (Thammasirirak et al. 2014).

On the other hand, there are few reports of parasitic infections in crocodiles, which indicates that this animal may be naturally resistant to most parasitic infections; thus, it is important to evaluate the immunomodulatory effect of this extract in parasitic diseases. In addition, this extract has been marketed and has shown a protective effect as a complementary therapy in various human diseases, with beneficial effects similar to extracts obtained from humans and bovine colostrum. For this reason, the dialyzable leukocyte extract obtained from crocodile lymphoid tissue may provide a potential therapeutic option for inflammation-related diseases like toxoplasmosis.

Toxoplasmosis is a highly prevalent infection in humans caused by the intracellular parasite *Toxoplasma gondii* that can be fatal in immunocompromised individuals (Tenter et al. 2000), causing severe damage to organs such as the ileum, spleen, liver, lung and brain (Schreiner & Liesenfeld 2009, Unno et al. 2013). The defence against the accelerated replication of the parasite and consequent severe tissue damage depends largely on the cellular immune response of Th1 cells. Thus, the aim of this study was to evaluate the effect of a DLE derived from crocodile lymphoid tissue (DLEc) on the parasite load and histopathology in the ileum, liver, spleen and brain in NIH mice infected with *T. gondii* (ME49 strain).

## MATERIALS AND METHODS

### Parasite

The ME49 (cyst-forming type II) strain of *Toxoplasma* was orally administered to NIH mice in the form of brain cysts every six to eight weeks.

### Study animals

We used female mice of the NIH strain, with an average weight of 24 grams, obtained from Laboratorios de Biológicos y Reactivos de México, Inc. (BIRMEX, S.A. de C.V.). Each experimental group consisted of 15 mice, which were kept in polysulfone cages with clean sawdust. Mice were provided with commercial food pellets (Rodent Chow 5001^®^; PMI Nutrition International, LLC) and drinking water *ad libitum.* The study was approved by the Committee of Research Ethics of the Escuela Nacional de Ciencias Biológicas of Instituto Politécnico Nacional, which issued the certificate number CEI-ENCB- 005/2015.

### Dialyzable leukocyte extract

Developed in Laboratorio Derivados Biológicos Sanare, SA (DeBiSa). Obtained from crocodile lymphoid tissue *(Crocodylus moreletii).* Each unit contained 0.102 mg of protein (1 U for human consumption).

### Experimental design

The mice were randomly divided into four experimental groups: healthy control group (n = 9), which received phosphate-buffered saline (PBS) intraperitoneally (IP); infection control group (n = 15), which received *T. gondii* cysts of the ME49 strain orally; DLEc control group (n = 15), which received the leukocyte extract intraperitoneally; and problem group (n = 15), which were infected with *T. gondii* cysts of the ME49 strain and treated with DLEc.

Infection was induced by administering 25 cysts per mouse orally; the cysts were obtained from brain homogenate of NIH mice that had been infected with the ME49 strain of *T. gondii* for at least eight weeks. Tissue cysts were counted in 10 μL of the brain suspension using light microscopy.

### Treatment scheme with DLEc

A total of 14 doses of the extract (each with 35 ng in a total volume of 100 mL of PBS per mouse) were intraperitoneally administered over eight weeks post-infection on days 2, 3, 4, 6, 8, 10, 13, 16, 23, 30, 37, 44, 51, and 58. Treatment was initiated 48 h after the parasite challenge.

The therapeutic dose was calculated based on 0.102 mg extract/70 Kg body weight, so the required amount administered was 35 ng extract per 24-25 g mouse.

### Histopathology

Five mice from each experimental group and three from the healthy group were sacrificed to assess histopathology at weeks 1, 4 and 8 post-infection. The sacrificed mice had their ileum, spleen, liver and brain (the latter only from mice sacrificed at week 8) removed and fixed in formaldehyde (10% in PBS) at pH 7.2 for subsequent dehydration and paraffin embedding. Slices of 5 μm were prepared and stained with haematoxylin-eosin (H&E).

### Determination of the parasite load

At weeks 4 and 8 post-infection, 5 mice in the infection control group and 5 mice in the infected group treated with DLEc were sacrificed, all according to NOM-062-ZOO-1999. The whole brain mass was removed, weighed and macerated in 2 mL of sterile PBS using a tissue homogeniser. The cysts were counted twice in 10 μL of the homogenate using light microscopy.

### Statistical analysis of the parasite load

Significant differences were determined with an unpaired Student's *t*-test using SPSS software v.15.0. Differences were considered significant when p < 0.05.

## RESULTS

### Ileal histopathology

**Fig. 1:**
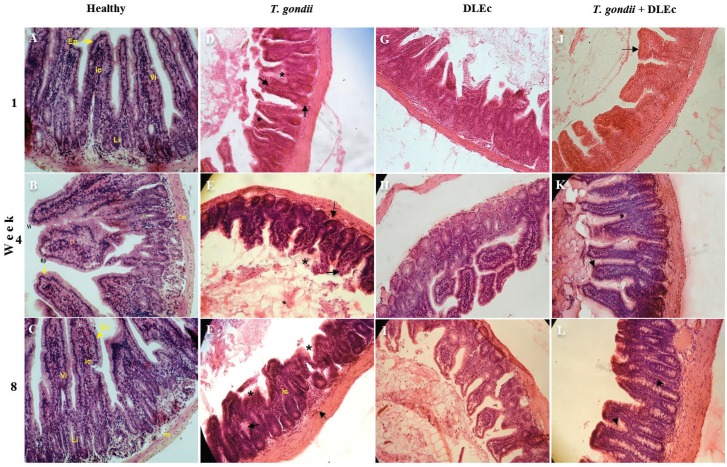
micrographs of histological changes in the ileum from NIH mice at weeks 1, 4 and 8 after oral infection with 25 cysts of *Toxoplasma gondii* and treatment with Dialyzable leukocyte extracts from crocodile lymphoid tissue (DLEc). A-C, healthy control group (100x); D-F, infection control group, with fusion and loss of villi (*), vascular congestion and inflammatory cell infiltration (arrows); G-I, DLEc control, slightly shortened villi; J-L, infection group treated with DLEc, showing recovery of the structure of the villi (arrow) and increased number of goblet cells (arrowhead). H&E; 20x. Ep: epithelium; ic: inflammatory cells; Li: Lieberkuhn's glands; m: muscle layer; Vi: intestinal villous.

In the first four weeks after infection, the mice infected with *T. gondii* showed fusion and destruction of villi; loss of continuity of the epithelium; increased infiltration of inflammatory cells into the corium, lamina propria and muscular layer; vascular congestion and haemorrhagic foci. Shortening of the villi was also observed at week 8. The mice treated with DLEc showed no significant histopathology, with intact and continuous epithelium and only a slight shortening of villi from the first week of treatment. The infected mice treated with DLEc showed recovery in the structure of the villi and epithelial lining, as well as fewer inflammatory foci, fewer fused villi, decreased vascular congestion and an increased number of goblet cells. The healthy control group had normal anatomical structures, intact villi and simple columnar epithelium (Fig. 1).

### Liver histopathology

The infected mice showed significant pathology at week 4 post-infection: generalised infiltration of inflammatory cells, loss of continuity of the hepatocyte cords, picnosis, vascular congestion and free tachyzoites.

**Fig. 2:**
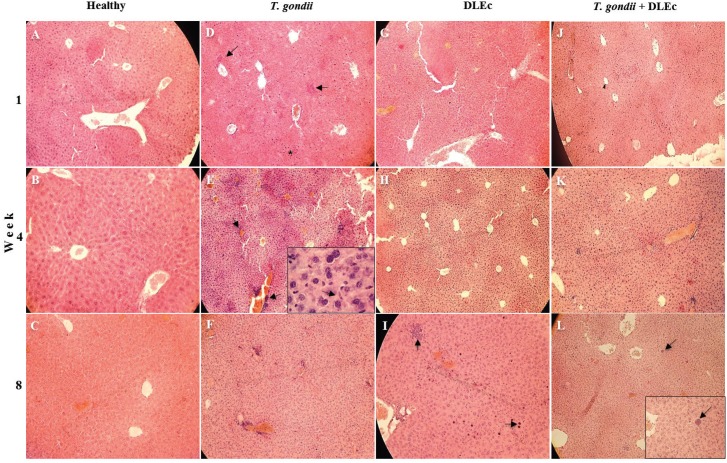
micrographs of histological changes in the liver from NIH mice at weeks 1, 4 and 8 after oral infection with cysts of *Toxoplasma gondii* and treatment with Dialyzable leukocyte extracts from crocodile lymphoid tissue (DLEc). A-C, healthy control group; D-F, infection control group showing abundant infiltration of inflammatory cells (arrows), vascular congestion and free tachyzoites (inset in Fig. E, arrow); G-I, DLEc control showing inflammatory cells (arrows); J-L, infection group treated with DLEc showing tissue recovery and the presence of *T. gondii* cysts with bradyzoites at week 8 (inset in Fig. L). H&E 10x, 60x.

The anatomical characteristics of the liver were preserved in mice treated with DLEc during the evaluation time. From week 4 onward, inflammatory cells were observed to be homogeneously distributed throughout the parenchyma (Fig. 2H), remaining until week 8 post-infection, which showed small groups of inflammatory cells (Fig. 2I, arrowhead).

The infected mice treated with DLEc showed light and homogeneous cell infiltration into the parenchyma from the first week, increasing greatly by week 4; the parenchyma remained without vascular congestion or perivascular infiltration of inflammatory cells (Fig. 2J-K). A trend of recovery in the liver tissue was observed at week 8 in all experimental groups; notably, the group of infected mice treated with DLEc had hepatocyte cords without apparent damage, fewer inflammatory foci and absence of vascular congestion, despite the presence of *T. gondii* cysts (Fig. 2L). The healthy control group presented anatomical integrity of hepatocyte cords, blood and sinusoid vessels.

### Spleen histopathology

**Fig. 3:**
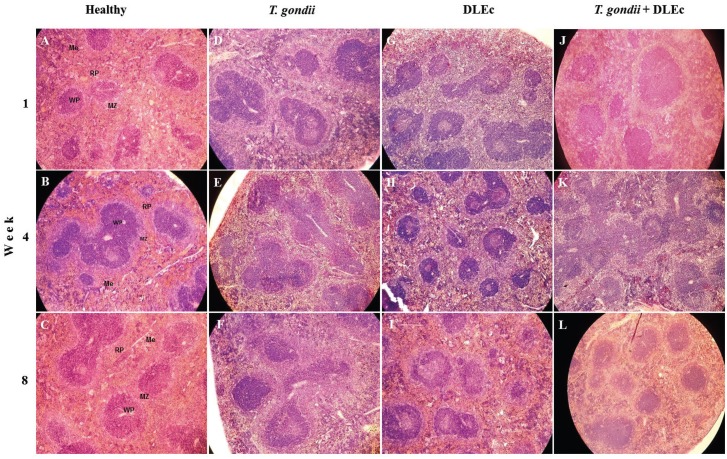
micrographs of histological changes in the spleen from NIH mice at weeks 1, 4 and 8 after oral infection with 25 cysts of *Toxoplasma gondii* and treatment with Dialyzable leukocyte extracts from crocodile lymphoid tissue (DLEc). A-C, healthy control group. D-F, infection control group showing lymphoid follicles of varying size and irregular shape; G-I, DLEc control group showing a greater number of lymphoid follicles, larger germinal centres and infiltration of megakaryocytes; J-L, infection group treated with DLEc showing a greater number of lymphoid follicles and heterogeneous marginal zone. In this group, some follicles do not have a germinal centre, and there is a large number of megakaryocytes compared with the infection control group. H&E; 10x. Me: megakaryocytes; MZ: marginal zone; RP: red pulp; WP: white pulp.

Mice infected with *T. gondii* showed enlarged lymphoid follicles of variable size and shape during the course of infection (Fig. 3D, F), some without a germinal centre and with a reduced marginal zone. The red pulp showed mild and diffuse congestion, infiltration of megakaryocytes and necrotic foci. The mice treated with DLEc showed a greater number of relatively smaller lymphoid follicles, as well as a reduced marginal zone, compared to those observed in the control group (Fig. 3H); however, follicles with prominent germinal centres and a heterogeneous marginal zone (Fig. 3I) were observed at week 8. The red pulp showed abundant lymphocytes and megakaryocytes. The group of infected mice treated with DLEc showed follicles with an increased number of interfollicular cells in the first four weeks after infection. At week 4, it was possible to observe the fusion of follicles with heterogeneous marginal zones (Fig. 3J-K); at week 8 post-infection, tissue recovery was observed, with well-structured lymphoid follicles, germinal centres and marginal zones with normal appearance and size. The red pulp showed a large infiltration of lymphocytes and megakaryocytes. The healthy control group showed well-defined white and red pulp (Fig. 3A, C).

### Brain histopathology

**Fig. 4:**
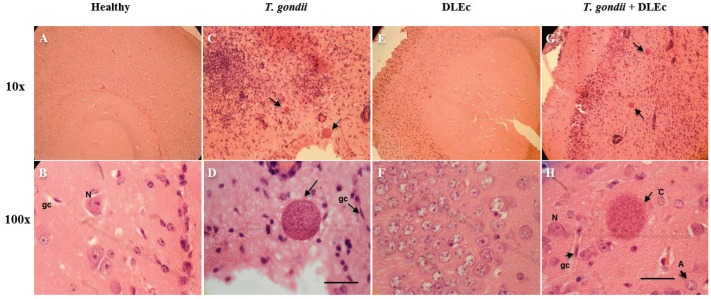
histopathology in the brains of healthy mice, mice infected with *Toxoplasma gondii* and mice treated with Dialyzable leukocyte extracts from crocodile lymphoid tissue (DLEc) at week 8 post-infection. Both the healthy control group (A and B) and the DLEc control group (E and F) show normal anatomic structure. The brains of the mice in the infection control group (C and D) and the infection group treated with DLEc (G and H) show perivascular inflammatory cells, endothelial hypertrophy, gliosis and tissue *T. gondii* cysts with bradyzoites (arrows). However, the group treated with the extract showed a milder inflammatory process compared to the infection control group. H&E; 10x, 100x. Bar: 30 μm; A: astrocyte; c: cysts; gc: glial cell; N: neuron.

The brain of mice in the healthy control group (Fig. 4B) and in the group inoculated with DLEc (Fig. 4E-F) both showed a normal structure without obvious abnormalities, molecular and granular layers with granular cells, neurons and glial cells. In contrast, the brains of infected mice (Fig. 4C-D) showed perivascular mononuclear infiltration, glial nodules and tissue cysts with well-defined walls (Fig. 4D). The brains of infected mice treated with DLEc (Fig. 4G-H) showed focal gliosis and tissue cysts with diffuse walls; the inflammatory process was milder, and tissue structure was preserved.

### Parasite load in the brain

**Fig. 5:**
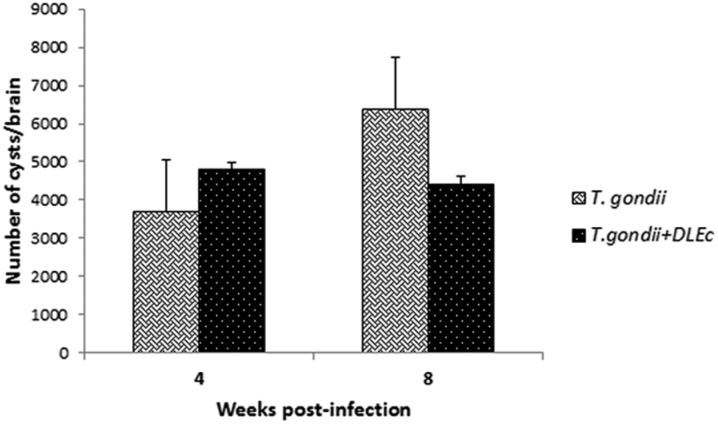
effect of treatment with DLEc on parasite load at weeks 4 and 8 post-infection in NIH mice (n = 5) infected with *Toxoplasma gondii* cysts (ME49 strain). Protection was evaluated by counting the number of cysts per brain. The values represent the mean ± SD of 2 independent experiments; the differences were not statistically significant (p > 0.05).

At week 4, the infection control group showed an average parasite load of 3692 cysts, while the group treated with DLEc had an average load of 4804 cysts. At week 8, the parasite load of the control group increased to 6383 cysts, while the parasite load of the treated group decreased to 4415 cysts. These results show a reduction of 31% in the parasite load of the group treated with DLEc compared with the infection control group (Fig. 5); however, the difference was not statistically significant.

### Survival

**Fig. 6:**
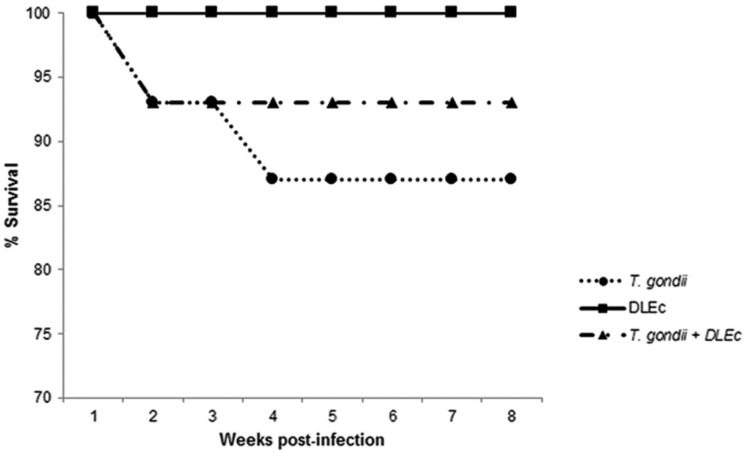
effect of treatment with Dialyzable leukocyte extracts from crocodile lymphoid tissue (DLEc) on mortality in NIH mice orally infected with *Toxoplasma gondii* cysts of the ME49 strain. Mice received a total of 14 doses (each with 35 ng) of the extract, administered intraperitoneally over eight weeks after infection on alternate days. Fifteen mice were used for each group.

Fig. 6 shows that the survival rate of mice infected with *T. gondii* was 87%, while the survival rate of mice treated with the extract was 93%.

## DISCUSSION

NIH mice are laboratory rodents that best represent the genetic variability in a typical population, so they can simulate the heterogeneity of the human population and thus be used to evaluate the effect of DLEc under these conditions. In addition, these rodents have been used as an alternative murine model in pharmacological and immunological studies of some parasitic infections, including toxoplasmosis (Martínez-Gómez et al. 2009, 2011).

The acute phase of *Toxoplasma* infection involves homeostatic imbalance in the intestine that causes severe ileitis combined with dysbiosis in the intestinal microbiota, which exacerbates the inflammatory response (Egan et al. 2012). In our model, the group of mice treated with DLEc preserved the integrity of the epithelial lining of the intestinal villi, and there was an increased number of goblet cells. These conditions are likely to favour an increase in secretory IgA levels and mucus production, which we suppose may prevent the parasite from penetrating the intestinal mucosa and spreading throughout the system.

Histopathological analysis showed that infected mice that were not treated with the extract had severe tissue damage during the acute phase of infection, with a large number of inflammatory foci, vascular congestion, necrosis and cell lysis in ileum and liver. This coincides with findings reported by Liesenfeld et al. (1996) and Egan et al. (2012). Furthermore, there were changes in the size and shape of the white pulp of the spleen, as described by Zenner et al. (1999) and Glatman-Zaretski et al. (2012). The brains showed evidence of inflammatory infiltration, gliosis and the presence of *T. gondii* cysts; these are important pathological characteristics of the chronic phase of infection (Gulinello et al. 2010, Parlog et al. 2015).

It is known that toxoplasmosis is lethal in murine models, with critical inflammatory damage from the onset of infection. This inflammatory phenomenon is associated with the participation of proinflammatory cytokines, such as IL-17, that are involved in the development and recruitment of neutrophils; a low number of neutrophils can reduce IL-12 production, favouring parasite replication and possible death of the host (Kelly et al. 2005). Neutrophils are also involved in the phagocytic activity of macrophages through lytic enzyme activity and the increased release of hydrolases and other enzymes, which together contribute to the limitation of tachyzoites in different organs.

The group of mice infected with *T. gondii* showed changes in the tissue structure of the organs studied. Furthermore, we observed the presence of free tachyzoites in the liver parenchyma (week 4). Tachyzoites probably attracted a greater number of inflammatory cells and, when activated, exacerbated the inflammatory response, causing cell lysis; this effect combined with the mechanical damage produced by the tachyzoites.

The mice treated with DLEc showed less tissue damage, fewer inflammatory foci and the absence of free tachyzoites. However, the spleen showed a reduction in the marginal zone at week 4, probably due to a decreased number of lymphoid and mononuclear cells, which reduces protection against the parasite and causes an increased parasite load in the brain. Nevertheless, in the eighth week, there were lymphoid follicles with well-defined marginal zones, suggesting the involvement of B lymphocytes to control the infection and reduce the parasite load.

Based on the studies by Fabre et al. (2004) that examined inflammatory processes associated with tissue damage - specifically the modulation of pro- and anti-inflammatory cytokines and increase of iNOS produced by DLE – we assume that the DLEc regulates the production of these cytokines and nitric oxide, increasing the activation of neutrophils and macrophages. These cells contribute to the control of infection and maintenance of tissue integrity, thus forcing the parasite to become a cyst or be eliminated by cytotoxic mechanisms.

At the brain level, it was observed in week 4 that the infection in the control group showed lesions similar to those described by Ferguson et al. (1991), as well as the presence of the characteristic cysts of *T. gondii.*

These alterations are due to a dysfunction in the blood-brain barrier, caused by parasite molecules that increase the permeability of the barrier and facilitate the establishment of the parasite. Astrocytes and glial cells play an important role in the protection against infection by secreting cytokines such as IL-1, IL-6, GM-CSF, IL- 10, IFN-g, and chemotactic cytokines (IP-10 and MCP- 1), which prevent lesions and restrict the replication of the parasite (Wilson & Hunter 2004, Drögemüller et al. 2008, Strunk et al. 2014). When the parasite infects a large number of these cells, their protective effect is diminished or lost, which aggravates tissue damage and the inflammatory process, favouring the formation of tissue cysts. In our model, the group of mice treated with DLEc showed fewer inflammatory foci, a slight increase in glial cells and a reduction of the parasite load at week 8; this suggests that the intraperitoneal administration of DLEc in mice triggers a nonspecific immune response, regulating not only the levels of pro- and anti-inflammatory cytokines, as mentioned above, but also of chemotactic molecules and ON, which control parasite replication.

Although the inflammatory phenomenon is apparently highly destructive, it ensures that all immunological elements involved in restricting the active replication of the parasite work in favour of the host, increasing the survival rate of infected animals. This study showed that DLEc allows the inflammatory phenomenon in the intestine, liver, spleen and brain to occur in a regulated manner, so that there is less tissue destruction compared to the infection control group; moreover, the survival rate of the animals treated with DLEc is higher compared to the infection control group.

This indicates that hosts that receive DLE can generate a well-directed immune response against pathogens such as *T. gondii,* which are susceptible to the effects of the TH1 immune response. This immune response involves pro-inflammatory cytokines, IFN-g, activated macrophages and related events such as the release of oxygen and nitrogen metabolism products, which may decrease parasite replication (Merchant et al. 2006, Gaddi & Yap 2007, Franco-Molina et al. 2008, Thammasirirak et al. 2014, Theansungnoen et al. 2014).

### In conclusion

Administration of DLE derived from crocodile lymphoid tissue had a modulating effect on the ileum, liver, spleen and brain of NIH mice infected with *T. gondii,* reducing tissue damage and the parasite load associated with the inflammatory process, which makes it a potential adjunctive therapy in the conventional treatment of toxoplasmosis.
